# 3D-Printed Alginate-Based Hydrogels with Appropriate Rheological Properties and Efficient Development of Cell Spheroids

**DOI:** 10.3390/polym17131730

**Published:** 2025-06-21

**Authors:** Alida Mazzoli, Stefania Greco, Francesca Luzi, Maria Caterina Evangelisti, Abel Duménigo González, Valeria Corinaldesi, Manila Caragiuli, Marco Rallini, Debora Puglia, Saverio Cinti, Paolo Moretti, Luigi Torre, Pasquapina Ciarmela

**Affiliations:** 1 Department of Science and Engineering of Matter, Environment and Urban Planning (SIMAU), Università Politecnica delle Marche, UdR INSTM, Via Brecce Bianche, 60131 Ancona, Italy; m.c.evangelisti@pm.univpm.it (M.C.E.); v.corinaldesi@staff.univpm.it (V.C.); 2 Department of Experimental and Clinical Medicine, Università Politecnica delle Marche, Via Tronto 10/A, 60121 Ancona, Italy; s.greco@staff.univpm.it (S.G.); a.dumenigo@pm.univpm.it (A.D.G.); s.cinti@staff.univpm.it (S.C.); p.ciarmela@staff.univpm.it (P.C.); 3 Department of Industrial Engineering and Mathematical Sciences (DIISM), Università Politecnica delle Marche, Via Brecce Bianche, 60131 Ancona, Italy; m.caragiuli@staff.univpm.it; 4 Department of Civil and Environmental Engineering, University of Perugia, UdR INSTM, Strada di Pentima 4, 05100 Terni, Italy; marco.rallini@unipg.it (M.R.); debora.puglia@unipg.it (D.P.); luigi.torre@unipg.it (L.T.); 5 Center of Obesity, United Hospitals—University of Ancona, 60020 Ancona, Italy; 6 Dipartimento di Scienze della Vita e dell’Ambiente (DISVA), Università Politecnica delle Marche, Via Brecce Bianche, 60131 Ancona, Italy; paolo.moretti@staff.univpm.it

**Keywords:** bio ink, cell growth, cell laden hydrogel, 3D bioprinting, regenerative medicine, rheology

## Abstract

In the last years, considerable innovation has been made regarding bioprinting, particularly in the development of cell-loaded hydrogels. The specific properties of the bioinks are crucial for printing an adequate cell-laden hydrogel structure. In this research, we aimed to develop a 3D-printable hydrogel using a natural biocompatible polymer. The process is based on the use of sodium alginate subjected to calcium ion cross-linking for immediate stiffness after printing. Using the Cellink INKREDIBLE+ printer (Cellink Inc., Goteborg, Sweden), 3D structures were successfully produced. The developed bioink exhibited a viscosity suitable for extrusion printing while ensuring its structural integrity at the same time. Next, 3D spheroids developed by using bioinks were morphologically characterized by using light, a fluorescent microscope, and field emission scanning electron microscopy (FESEM). In conclusion, the properties of the construct obtained using the lab-formulated biocompatible polymer hydrogel suggest its potential use as a framework for three-dimensional cell culture, with possible applications in both fields of research and regenerative medicine.

## 1. Introduction

Over the past ten years, 3D bioprinting has emerged as a groundbreaking technology in tissue engineering, making remarkable progress in the reconstruction of transplantable tissues and also intricate organs, such as the human ear, bones, skin, and nose. This approach, which integrates additive manufacturing (AM), biology, and material science, entails the deposition of bioinks (viscous substances infused with living cells and supplementary matrix elements) via 3D printers to accomplish layer-by-layer assembly for fabricating three-dimensional structures through computer-aided design (CAD).

Several bioprocessing strategies, including selective laser sintering (SLS), inkjet printing, and extrusion/deposition-based methodologies, have been devised to facilitate the 3D printing of viable cells and biological scaffolds [1]. While significant advancements have been achieved in producing clinically sized hard tissues, such as bones, the fabrication of scaffolds for soft tissues is still constrained to small-scale medical applications with limited structural intricacy [2].

Recent advancements in 3D bioprinting research have delivered encouraging results, particularly in generating skin substitutes for individuals suffering extensive skin injuries caused by burns, chronic ulcers, cancer, or surgical procedures. While conventional tissue-engineering approaches encounter difficulties in replicating biomimetic and heterogeneous tissue structures, emerging 3D bioprinting technologies present a promising alternative.

Bioinks, composed of diverse blends of biomaterials, biomolecules, and cells, influence printability, biocompatibility, and mechanical stability. After bioprinting, constructs can undergo crosslinking to reinforce their form and structure, ensuring consistency, reproducibility, and precise modulation. These multi-component bioinks have been designed to facilitate the fabrication of biomimetic and intricate tissue structures [3].

Recently, bioinks have been characterized as material formulations compatible with automated biofabrication techniques, which must incorporate living cells [4]. Bioink is a blend of cells, biopolymers, and biologically active compounds that influence cell viability during printing, support cell multiplication and expansion, and promote tissue formation [5]. Bioinks designed for printing living cells are typically scaffold-based, where cells are encapsulated in hydrogels or comparable materials, offering adhesion sites to enable cell spreading.

The selection of an appropriate bioink is essential for achieving optimal bioprinting conditions [6]. Hydrogels are essential to maintaining the structural integrity and three-dimensional stability of printed tissues. A fundamental characteristic of hydrogels as bioinks is their ability to transition between liquid and solid states under specific conditions, enabling the printing process, as these transition parameters directly influence 3D bioprinting results.

Recent studies have concentrated on developing materials and bioink formulations with adequate rheological properties to preserve both dimensional stability and biocompatibility, fostering cell proliferation. Cells are essential components of bioinks, while carrier biomaterials are equally vital, as they assist in establishing a stable spatial arrangement, sustaining cellular viability during printing, and supporting post-printing proliferation and differentiation [7].

Despite the promising outputs, the industrial application of 3D bioprinting to produce customized, functional living constructs faces ethical barriers [1] and technical challenges, such as achieving high-resolution cell deposition and controlled cell distribution [3], that need exploration and identification before therapeutic 3D bioprinting can be widely adopted in human patients.

Bioinks pose another critical challenge due to their high cost and limited accessibility. In addition, the intricate nature of bioinks further contributes to the complexity and cost of the bioprinting process. Although many bioinks are commercially available, it may be necessary to obtain a personalized composition. Currently, to obtain the materials, it is necessary to purchase them from the same companies that supply the bioprinters. This fact greatly limits accessibility, as the purchase of materials is tied to specific suppliers, often associated with the biological printers owned by the same companies.

The small quantity and high prices of these materials are an additional obstacle, especially in the research field, where budgets can be tight. The purchase of high-cost bioinks can become difficult to sustain, limiting the scope of scientific experiments and investigations in the field of 3D bioprinting.

Addressing these issues is crucial to the implementation of 3D bioprinting in both research and therapeutic applications. Efforts to simplify and/or customize the manufacturing process, reduce costs, and establish more accessible bioink resources are critical to unlocking the potential of this technology. Overcoming these challenges will not only improve the viability of 3D bioprinting but also increase its impact in various fields, from research to regenerative medicine, and more.

In this study, we introduce lab-formulated alginate hydrogel structures. Alginate hydrogels are selected due to their extensive research and application in the medical field [8]. This choice is attributed to their known biocompatibility, controllable stiffness, and ability to form highly porous structures conducive to cell regeneration [2]. These properties make alginate suitable to produce biodegradable hydrogels utilized in the manufacturing of skin scaffolds.

Furthermore, the crosslinker steps performed by immersing the printed hydrogel in 80 mM calcium chloride significantly extended the stability of the printed polymeric system without compromising cell viability. The material has been chosen for its cost-effectiveness, widespread availability, and ease of processing.

To carry out this first study, we used cancer cells as they grow easily and are prone to form three-dimensional aggregates. Specifically, we used the leiomyosarcoma cell line (SK-LMS-1), which represents a malignant tumor of soft tissues and belongs to the sarcoma family. We chose these cells because we have already used them in previous biological studies and have already developed the method with the printer we currently have in our laboratory [9].

The rheological characteristics of hydrogels are analyzed before the printing process, and additionally, cell viability after printing is monitored over a defined timeframe. This research aims to design three-dimensional printable hydrogels derived from biocompatible natural polymers and assess their mechanical behavior and ability to support cell growth for potential applications in scientific studies.

## 2. Materials and Methods

### 2.1. Bioink Formulation and Characterization

In this study, a lab-formulated hydrogel has been formulated and compared with a commercial formulation of bioink provided by the same manufacturer of the bioprinter used in research activity (Cellink bioink, Göteborg, Sweden; https://www.cellink.com/product/cellink-bioink/ accessed on 12 May 2025). The lab-formulated hydrogel is based on sodium alginate (Sapore Puro, Gioia Group srl, Turin, Italy), dissolved in deionized water at room temperature. An 80 mM CaCl_2_ solution (Product number 223506 CaCl_2_ dehydrate, Merck Life Science Srl, Milan, Italy) was used as a cross-linking agent for sodium alginate. A range of sodium alginate concentrations of 2%, 3%, 6%, 8%, and 10% (*w*/*v*) were tested to determine the optimal hydrogel formulation with self-support ability. A content of 8% (*w*/*v*) alginate represented the lowest content required to obtain the interface layers. However, 10% (*w*/*v*) alginate was found to be the best concentration, taking into account the changed final alginate content when the cell suspension was added to the gel. Then, 10% (*w*/*v*) dry sodium alginate powder was dissolved in deionized water, slowly pouring the alginate into powder on the surface of the water and waiting for a few hours for a homogeneous hydrogel/solution. The hydrogel was maintained at 4 °C until use and brought to room temperature before printing.

#### 2.1.1. Rheological Tests

The rheological properties of sodium alginate hydrogels at 8 and 10% (*w*/*v*) were investigated at 25 °C (temperature of bioprinting process) by using an MCR 702e Anton-Paar rheometer and compared with the behavior of a commercial bioink (Cellink bioink, Göteborg, Sweden; https://www.cellink.com/product/cellink-bioink/ accessed on 12 May 2025). Tests were performed by using plane–plane geometry (gap of 1 mm diameter of the upper plate of 25 mm). Oscillatory shear analyses were executed as a function of the oscillation frequency (ω) in the range of 0.1–100 rad/s for a constant strain (γ) of 0.1% within the linear range of viscoelasticity. The loss tangent measured as tanδ = G″/G′ provides information about the viscoelastic properties of the sample (for elastic solids tanδ < 1, while, for viscous fluids, tanδ > 1). The viscosity (η) was determined as the ratio between shear stress τ (in Pa) and the shear rate $\dot{\gamma }$ (in s^−1^) in stationary shear flow conditions, for $\dot{\gamma }$ varying in the range of 0.01–1000 s^−1^.

#### 2.1.2. Cell Lines

Leiomyosarcoma cell lines (SK-LMS-1) were furnished from ATCC (RRID: CVCL_0628, American Type Culture Collection, Manassas, VA, USA). The cells were maintained in low-glucose DMEM (Corning, New York, NY, USA) supplemented with 10% FBS (Euroclone, Milan, Italy) and 1% P/S antibiotic (Euroclone, Milan, Italy). Incubation was carried out at 37 °C in an atmosphere of 95% air and 5% CO_2_.

### 2.2. Bioink Preparation

To prepare the bioink mixture, cells previously detached from a T75 flask (Corning, New York, NY, USA) were treated with trypsin-EDTA (Corning, New York, NY, USA) and quantified using the LUNA II automated counter (Logos Biosystem, Annandale, VA, USA). The cells were then centrifuged at 250 rpm for 5 min to form pellets. Following the manufacturer’s guidelines provided by Cellink (Cellink Inc., Göteborg, Sweden), the cell suspension was combined with the gel for both bioinks using the following concentrations: 100 µL of cell pellets were mixed with 1 mL of bioink, resulting in a final bioink formulation with a cell suspension containing 5 × 10^5^ cells per mL.

#### 2.2.1. Bioprinting

INKREDIBLE+ bioprinter (Cellink Inc., Göteborg, Sweden), a pneumatic-based extrusion bioprinter (pressure range from 5 to 400 kPa) with a dual-extruder for bioprinting hydrogels, was used. Its design allows it to easily fit into a standard biosafety cabinet, enabling operation under sterile conditions. The “+” model possesses a digitally controlled heating unit (with a temperature range of 25–85 °C) and pneumatic actuated dual-extruder heads. INKREDIBLE+ operates with Repetier-Host software version 2.3.2, which runs G-code, and has a plotting resolution of 10 µm in the X-axis and Y-axis (horizontal plane) and 100 µm in the Z-axis (layer resolution).

#### 2.2.2. Bioink Printing Process

The INKREDIBLE+ 3D bioprinter was used for hydrogel printing. During printing, the monitoring was performed through the Cellink proprietary software DNA Studio 4. Utilizing a three-dimensional CAD model (Computer-Aided Design), which is converted into coordinates through a geometric programming language (G-code), the matrices were printed following the selected CAD design in a (10 × 10) mm^2^ grid within 6-well plates (Corning, New York, NY, USA). The chosen geometry allowed the cells to form the predicted spheroids. The extrusion syringes are connected to an adjustable pressure regulator. The hydrogel deposition flow has been fine-tuned by modifying the extrusion pressure and the Z-axis distance between the nozzle tip and the printing surface. Six square scaffolds were printed on a six-well plate using a 22 G nozzle (with an opening of 410 µm). Table 1 presents the printing parameters, selected for the lab-formulated bioink, in order to optimize the extrusion-based 3D printing.

#### 2.2.3. Post Printing Crosslink

After printing, the scaffolds were subjected to a chemical polymerization process using 80 mM calcium chloride CaCl_2_ for 15 min. This polymerization was implemented on the printed matrix. Subsequently, the polymerized networks were washed with PBS (Corning, New York, NY, USA), immersed in the growth medium of SKLMS-1 cells, and subsequently placed in an incubator with 5% CO_2_ and at 37 °C.

#### 2.2.4. Mechanical Properties

Compression tests of post-crosslinked sodium alginate hydrogels at 8% and 10% (*w*/*v*) were performed at 25 °C using an MCR 702e Anton Paar rheometer. Hydrogel discs with a diameter of 25 mm were tested employing a flat probe of 25 mm diameter, with a displacement rate of 0.1 mm/s. Compressive stress was evaluated at 50% strain, corresponding to a total displacement of 1 mm.

#### 2.2.5. Degradation Performance In Vitro

In vitro degradation tests were performed in low-glucose DMEM (Corning, New York, NY, USA) at 37 °C. Hydrogel scaffolds (10 × 10 × 1) mm^3^ were immersed in 7.5 mL of DMEM and incubated at 37 °C. At predetermined time points (2 and 6 days), the hydrogel discs were collected, washed with deionized water, and dried in an oven at 37 °C for 48 h until reaching a constant weight. For each time point, three specimens were tested. Finally, the weight of the dried discs was recorded, and the percentage of the remaining weight was calculated using the following equation (Equation (1)):

(1) $$Remaining\;weigth\;\left(\%\right)=\frac{Dry\;weigth\;at\;selected\;time\;pioints}{Dry\;weith\;at\;time\;0}\times 100$$

### 2.3. Scaffold Characterization

#### 2.3.1. Optical Microscope Observations

The first morphological evaluation performed on the scaffolds with cells was performed using the inverted phase contrast microscope (Nikon Eclipse E600, Nikon Corporation, Tokyo, Japan). The observation was carried out after 24 h, i.e., the day after printing, after 48 h, and after 5 and 6 days. At the same time, images were acquired to evaluate the growth of the spheroids formed.

#### 2.3.2. Statistical Analysis

All statistical analyses were performed using GraphPad PRISM 9 (version 9.5.0). The results are presented as minimum/maximum box whisker plots, with the median value represented by a solid horizontal line within each box. To assess differences across various time points in the variance analysis, a non-parametric ANOVA test was applied. For multiple comparisons, the Kruskal–Wallis test was conducted. Asterisks (*) denote statistical significance as follows: * *p* < 0.05; ** *p* < 0.01; *** *p* < 0.001; **** *p* < 0.0001.

#### 2.3.3. Dapi Staining

For 4′,6-diamidino-2-phenylindole (DAPI) staining (Thermo Fisher, Waltham, MA, USA), sterile PBS (Corning) was used to dilute the solution to a final concentration of 300 nM. Initially, the scaffolds were rinsed 1–3 times with sterile PBS before being coated with the fluorescent DAPI solution (300 nM) and incubated for 1–5 min, shielded from light. Finally, the staining solution was removed by washing the samples 2–3 times with PBS, and images were captured using a Nikon fluorescence phase-contrast microscope.

#### 2.3.4. Haematoxylin and Eosin

The scaffolds were fixed with 4% paraformaldehyde (Sigma-Aldrich; Merck, Darmstadt, Germany) and embedded in paraffin. The paraffin sections were rehydrated using xylene and a decreasing gradient of ethyl alcohol concentrations. After rinsing with 50% alcohol, the sections were immersed in distilled water for five minutes. They were then stained with hematoxylin (Bio-Optica, Milan, Italy) for two minutes, washed in distilled water, followed by eosin staining (Bio-Optica) for another two minutes, and rinsed again with distilled water. Finally, the samples were dehydrated through an increasing gradient of ethyl alcohol and xylene before being mounted with Eukitt Solution (Orsatec GmbH, Kindler GmbH & Co., Bobingen, Germany).

#### 2.3.5. Field Emission Scanning Electron Microscopy (FE-SEM)

Scaffolds (2 mm thick) were preserved in 2% glutaraldehyde-paraformaldehyde overnight at 4 °C. They were then post-fixed in 1% osmium tetroxide (OsO_4_) in phosphate-buffered saline (PBS) for 60 min at 4 °C, rinsed with PBS, and progressively dehydrated using increasing concentrations of ethanol. Drying of the samples consists of incubation of the scaffold in increasing concentrations of hexamethyldisilane (HMDS, Sigma Aldrich) and decreasing concentrations of ethanol, until inclusion in 100% HMDS. Subsequently, the scaffolds were mounted on aluminum supports called stubs, which are equipped with carbon-coated adhesive discs on which the scaffolds were adhered and subsequently subjected to gilding, i.e., the sputtering gold coating. Finally, the morphological analysis of the scaffolds was performed using a field emission scanning electron microscope (FESEM) Supra 40 (Zeiss, Oberkochen, Germany), capturing micrographs at various magnifications with an accelerating voltage of 5 kV.

## 3. Results

### 3.1. Rheological Tests

**Figure 1:**
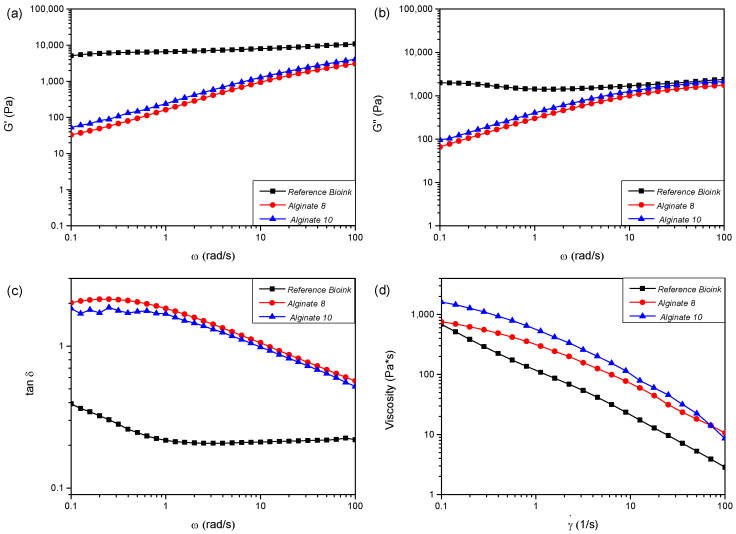
Curves of storage modulus (G′) (**a**), loss modulus (G″) (**b**), and loss tangent (tanδ) (**c**) as a function of frequency (ω) and viscosity (**d**) (η) as function of shear rate ($\dot{\gamma }$) for the reference “Bioink” and sodium alginate “Alginate” hydrogels.

The performances of the hydrogels can be characterized by measuring their rheological properties and evaluating different rheological characteristics, such as storage modulus (G′), loss modulus (G″), tanδ (loss tangent), and viscosity (η) [10]. High viscosity maintains the shape of the processed/extruded hydrogel, enhances mechanical stability, and supports the printing of multilayer structures. However, it may also lead to clogging of the injection port, causing uneven and discontinuous deposition of the bioink. In contrast, low viscosity can result in uneven cell distribution and improper cell deposition within the bioink. Furthermore, cells actively sense and respond to external mechanical stresses, adapting their behavior according to variations in the mechanical environment. Thus, the mechanical characteristics of the bioink used in bioprinting should be carefully considered, not only for the realization of complex structures but also for ensuring proper interactions between cells and the materials. Figure 1 shows results obtained in terms of viscoelastic parameters, such as G′ (Figure 1a), G″ (Figure 1b), and tanδ (Figure 1c) as a function of oscillation frequency, as well as viscosity (η) as a function of shear rate ($\dot{\gamma }$ hydrogel samples used as bioinks. Table 2 summarizes the rheological parameters detected for all the investigated bioinks at 25 °C. The values of storage modulus (G′) and loss modulus (G″) reported in Table 2 have been registered in correspondence with two different values of the angular frequency (ω): 1 rad/s and 100 rad/s. G′ and G″ measured for the bioink reference are higher with respect to the alginate-based system (Figure 1a,b). The values of storage and loss modulus for tested gels increase with increasing ω. Figure 1c shows the tanδ curves of commercial and alginate gels, highlighting the different behavior of tested samples. Reference bioink is characterized by elastic solid behavior (tanδ < 1), while alginate gels are viscous fluids (tanδ > 1) (below ω < 13 rad/s and ω < 10 rad/s for Alginate-8% and Alginate-10%, respectively).

**Figure 2:**
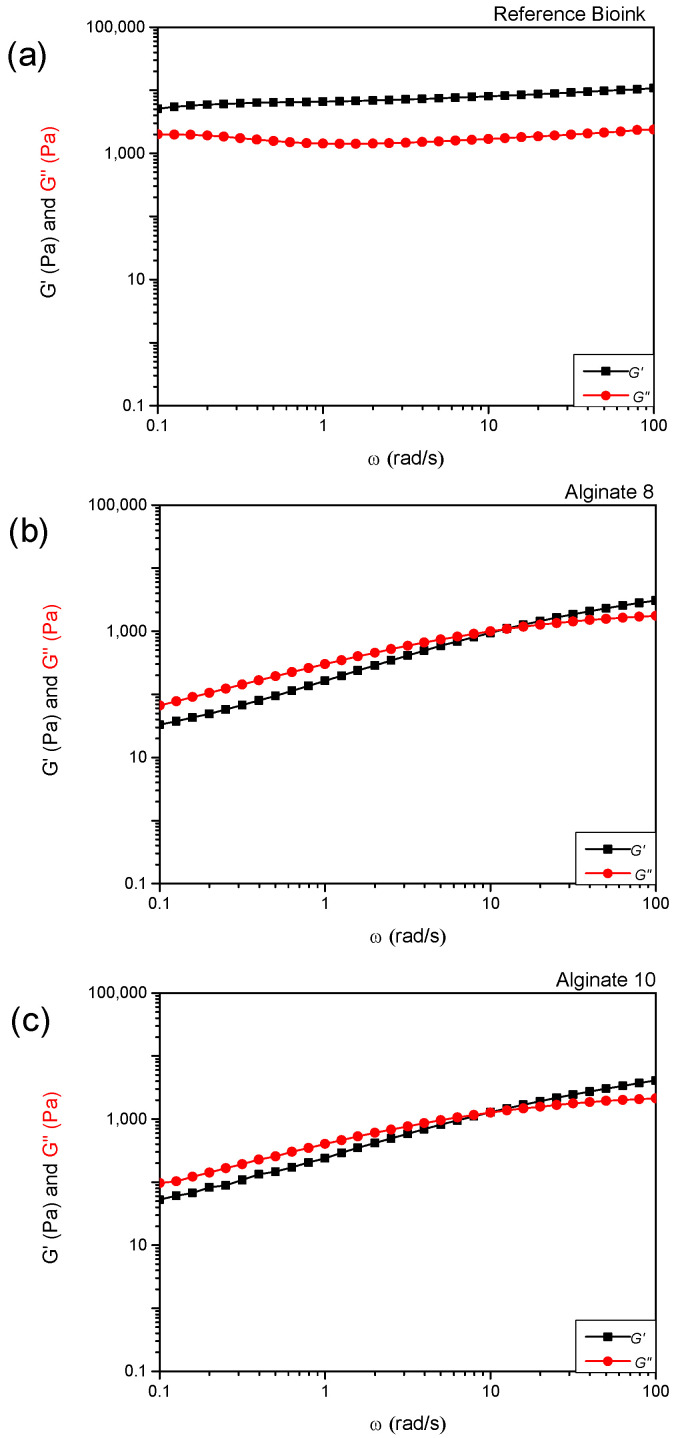
Values of storage modulus (G′) and loss modulus (G″) as a function of angular frequency (ω) for the reference “Bioink” (**a**) and sodium alginate “Alginate” hydrogels with different concentrations (8% (*w*/*v*) (**b**) and 10% (*w*/*v*) (**c**)).

Figure 2a shows the comparison of storage modulus (G′) and loss modulus (G″) values as a function of the angular frequency (ω) for the reference commercial product (labeled as “Bioink”). Materials with liquid-like behavior are considered unsuitable for the extrusion bioprinting process because they cannot maintain shape after extrusion and tend to lose the initial shape over time [11,12]. This limitation can be overcome by adding, immediately after the extrusion process, a cross-linker to allow the crosslinking process in polymeric scaffolds. In this experimental work, according to the literature, calcium chloride (CaCl_2_) was used as a cross-linker agent after the extrusion process of alginate-based materials [13].

### 3.2. Bioprinting

**Figure 3:**
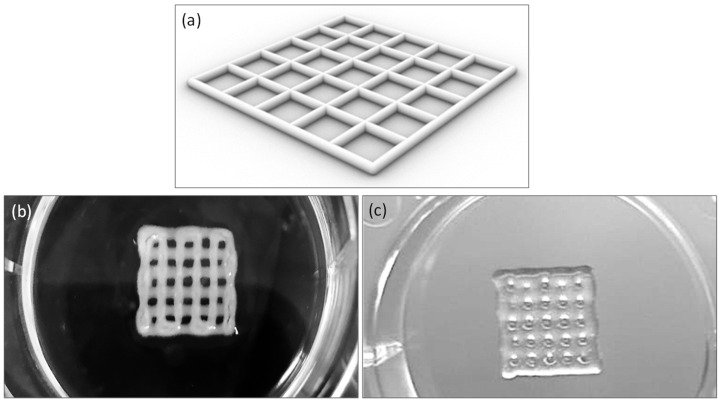
Printability of bioinks: square grid of scaffold model (**a**), 10 by 10 mm square grid printed with Cellink Bioink (**b**), lab-formulated bioink (alginate 10% *w*/*v*) (**c**).

The matrices were printed according to the model depicted in Figure 3a, utilizing the standard tessellation language (.STL) file format for 3D modeling. Specifically, the printed scaffolds consisted of two layers, each with a height of 0.4 mm, and had dimensions of (10.0 × 10.0 × 0.8) mm^3^, with a line spacing of 2 mm. Bioprinting was performed using both commercial alginate and lab-formulated alginate at a 10% *w*/*v* concentration, as depicted in Figure 3b,c.

### 3.3. Mechanical Properties

**Figure 4:**
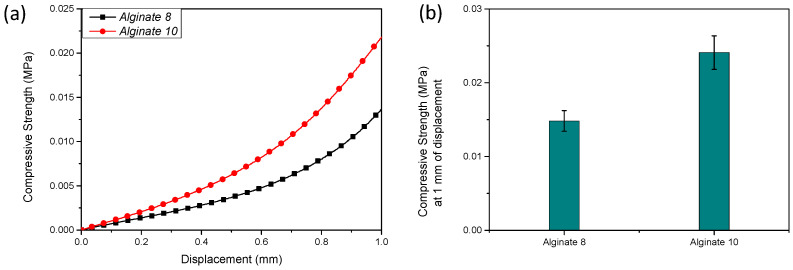
Compressive strength–displacement curves (**a**) and corresponding maximum compressive strength at 1 mm displacement (**b**) of sodium alginate (lab-formulated) hydrogels prepared at 8% (*w*/*v*) and 10% (*w*/*v*) concentrations.

Figure 4a,b illustrates the mechanical behavior of sodium alginate hydrogels. Specifically, Figure 4a shows the compressive strength–displacement curves, while Figure 4b reports the maximum compressive strength measured at a displacement of 1 mm. An increase in compressive strength was observed with an increasing sodium alginate concentration, highlighting the lower compressive strength of the 8% alginate formulation.

### 3.4. In Vitro Degradation Test

**Figure 5:**
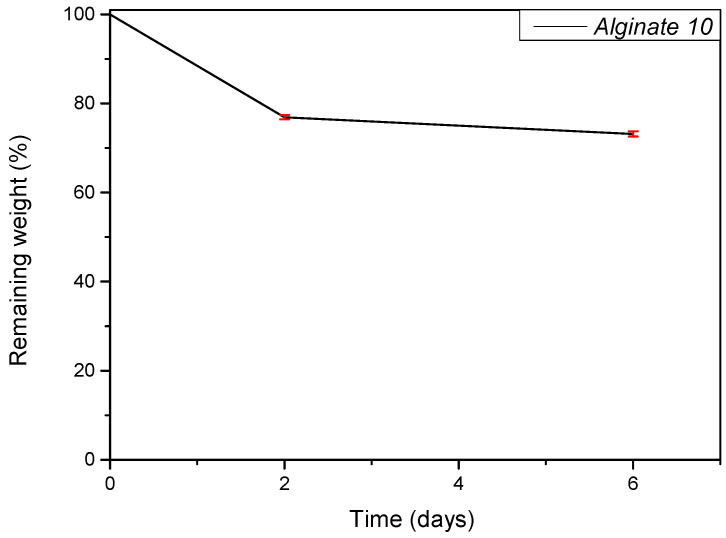
Hydrolytic degradation study. The percentage of remaining dry weight of Alginate 10% at different time points.

An in vitro degradation test was performed to evaluate the resistance of the scaffold in DMEM during cell culture. The test was conducted at different time points (0, 2, and 6 days). In this procedure, the hydrogels were incubated at 37 °C in a culture medium, and their degradation profile was monitored. Figure 5 presents the graph of the remaining weight. As shown, the recorded remaining weight values at both time points are comparable, indicating limited degradation over the observed period.

### 3.5. Morphological Investigation

The initial morphological assessment of cells cultured in a three-dimensional environment was performed over the subsequent days. Phase-contrast inverted microscopy was employed to monitor and quantify spheroid growth on the 10% alginate scaffolds. Observation of the Cellink alginate scaffold, however, was not possible due to its dense structure, which interfered with both visual inspection and image acquisition.

**Figure 6:**
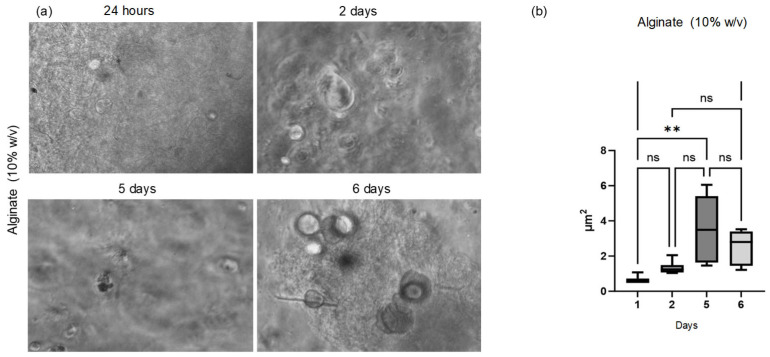
Optical observation and statistical analysis of spheroid growth. (**a**) Phase-contrast optical microscopy images of spheroids formed on the 10% *w*/*v* alginate scaffold at 24 h, 48 h, and 72 h and on days 5 and 6. (**b**) Quantitative analysis of spheroid area at days 1, 2, 5, and 6 (n = 6 biological replicates per time point, ~36 technical replicates per group). Statistical analysis shows a significant increase in spheroid area at day 5 (*p* = 0.0010) and day 6 (*p* = 0.0065) compared to day 1, indicating a positive cellular response and supporting the biocompatibility of the hydrogel. **: represents statistically significant differences for *p* < 0.05 and *p* < 0.01; ns: represents that there are no statistically significant differences.

For each time point (24 h, 48 h, 72 h, day 5, and day 6), morphometric analysis was conducted on a single 6-well plate (n = 6 biological replicates) (Figure 6). Approximately 6 images were acquired from each well, resulting in about n = 36 technical replicates per time point. The spheroid area was measured in each image, and the average area per well was calculated and used for statistical analysis. The spheroid area was determined from digital images acquired via phase-contrast microscopy and processed using ImageJ software version 1.54p (National Institutes of Health—NIH, Bethesda, MD, USA). Spheroids were manually identified and selected using the “Threshold” function to enhance edge detection, and the “Analyze Particles” function was then applied to calculate the area of each spheroid, expressed in square micrometers (µm^2^). The panels in Figure 4a illustrate spheroid growth from the day following printing up to day 6. The panels in Figure 4b show the results that indicate a positive cellular response and support the biocompatibility of the tested hydrogels.

### 3.6. Dapi Staining

**Figure 7:**
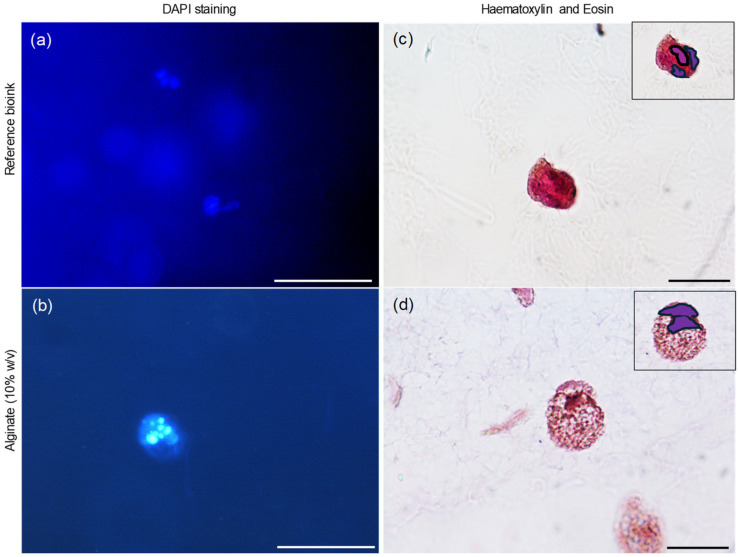
Morphological investigation. DAPI fluorescent staining of Cellink Bioink (**a**) and alginate (10% *w*/*v*) (**b**). Magnification 20×. Haematoxylin and eosin images of Cellink Bioink (**c**) and alginate (10% *w*/*v*) (**d**). The nuclei are highlighted in purple at the top left. Magnification 100×. Bar: 100 µm for magnifications 20× and 10 µm for magnifications 100×.

The morphological analysis was further extended using DAPI staining, which binds to the cells’ nuclei, enabling visualization of spheroids’ formation and, importantly, the quantification of the number of nuclei present, thereby facilitating morphological characterization. Following DAPI staining, the cells’ nuclei were clearly highlighted due to their fluorescence, confirming the cellular viability of the 3D aggregates. Figure 7a,b presents representative images of the staining for the Cellink alginate scaffold and the formulated hydrogel, respectively.

### 3.7. Haematoxylin and Eosin

Further analysis of the matrices was performed using hematoxylin and eosin (H&E) staining, followed by observation under an optical microscope. Hematoxylin, which stains nucleic acids, imparts a deep blue-purple color to the nuclei, while eosin stains proteins, resulting in varying shades of pink for the cytoplasm and extracellular matrix. H&E staining revealed that each spheroid was composed of multiple cells in both matrix types (Figure 7c,d), with the nuclei clearly highlighted in purple in the top left corner of the images.

### 3.8. Field Emission Scanning Electron Microscopy (FE-SEM) Observations

**Figure 8:**
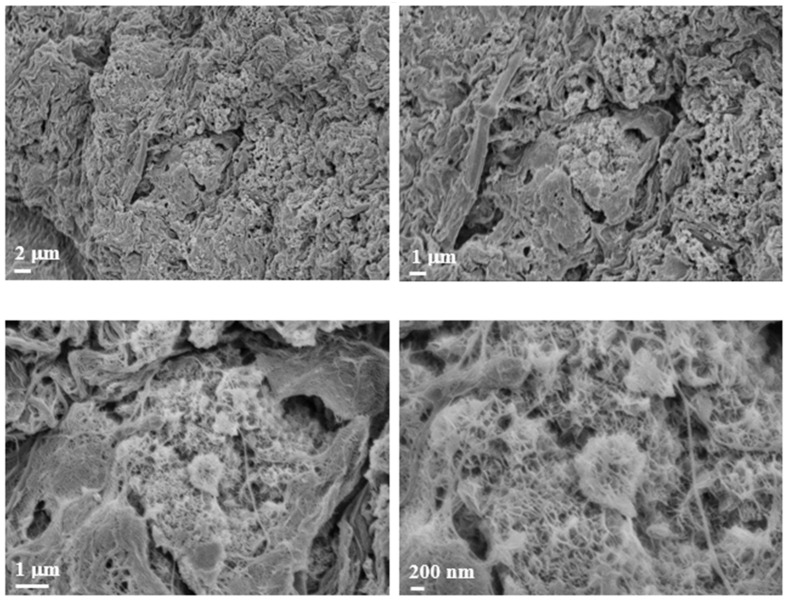
FE-SEM morphological investigation. Images of Cellink bioink alginate at different magnifications. The images show the presence of a biological structure indicated by the white arrowhead at different magnifications (2 µm, 1 µm, and 200 nm).

Following the promising results obtained with hematoxylin and eosin (H&E) staining, we further investigated the morphological characteristics of the scaffolds using Field Emission Scanning Electron Microscopy (FE-SEM). Figure 8 presents representative FE-SEM images of the commercial Cellink alginate scaffold. The figure shows the surface of a spheroid with a distinct disruption in the structure. This disruption allowed for a closer examination of the inner architecture, revealing a significant presence of mitochondria within the spheroid.

**Figure 9:**
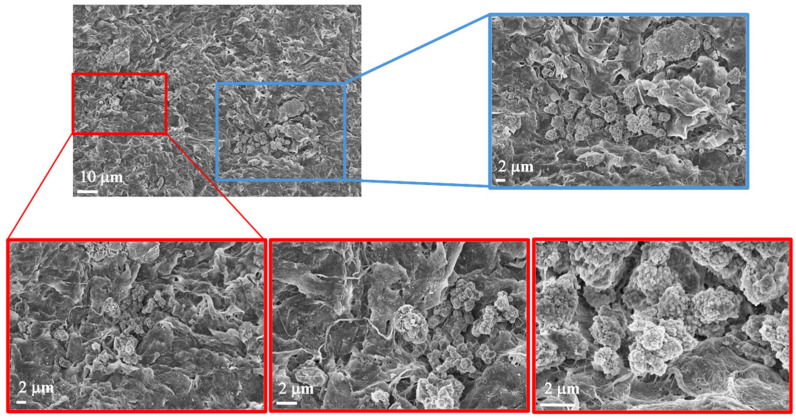
Morphological investigation using FE-SEM. Images of Cellink bionk alginate. The images show the presence of biological structures at different magnifications (10 µm and 2 µm).

**Figure 10:**
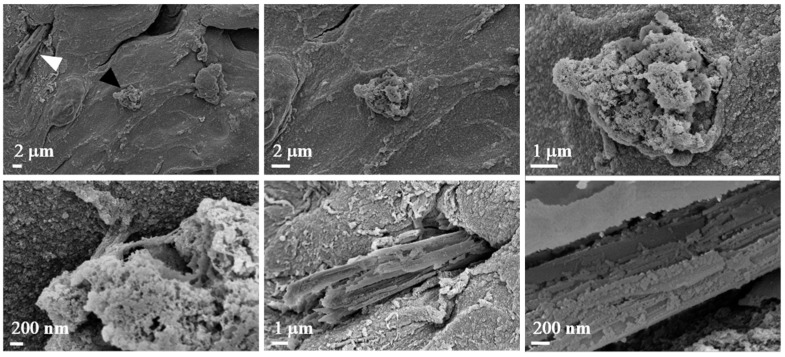
Morphological investigation using FE-SEM. Images of alginate (10% *w*/*v*). The images show the presence of biological structures at different magnifications (2 µm, 1 µm, and 200 nm).

In Figure 9, two panels are highlighted in the small enlargement, one in red and one in blue. The red panel displays three magnifications, each emphasizing the presence of mitochondria or, more generally, subcellular structures, given their small size. The blue panel, with its corresponding enlargement on the right, illustrates the surface of the spheroid, which also appears rich in mitochondria. Additionally, we observed filamentous structures that may resemble collagen fibers. However, it is important to note that the identification of these structures as collagen remains hypothetical, as it is based solely on morphological appearance. Further validation (e.g., through specific staining or biochemical analysis) would be required to confirm their nature. Nevertheless, collagen is known to constitute the extracellular matrix and play a crucial role in tumor formation and progression [5]. Figure 10 displays representative images of the 10% *w*/*v* alginate scaffold. These images reveal the presence of subcellular structures (denoted by the white arrowhead and captured at multiple magnifications) that, based on their small size, may correspond to mitochondria, as observed in the commercial matrix. Additionally, the surface of the spheroid exhibits fibril-like structures, which could potentially be collagen fibers (marked by the black arrowhead and shown at various magnifications). As previously mentioned, this interpretation remains speculative and would require further confirmation. These fibril-like features are particularly noticeable in the regions surrounding the spheroid, suggesting a possible interaction between the cells and the scaffold, possibly involving extracellular matrix deposition. Furthermore, the comparison between the two scaffolds highlights significant differences in their mesh structures. The 10% *w*/*v* alginate scaffold presents a more tightly organized mesh, while the commercial matrix displays a relatively looser, more interconnected network. These differences may influence the mechanical properties of the scaffolds and the behavior of the encapsulated cells, potentially affecting their proliferation and interaction with the surrounding environment.

## 4. Discussion

The rheological data obtained clearly underscore the suitability of the proposed bioink for bioprinting applications The detailed evaluation of its handling, elasticity, and viscosity characteristics demonstrated that the alginate-based bioink exhibits both liquid and solid behaviors. While its liquid form presents challenges during the bioprinting process, making it unsuitable for immediate printing, the use of a crosslinking agent in the post-printing stage effectively mitigates this issue, allowing the material to solidify and acquire the desired structural integrity. These findings suggest that a 10% *w*/*v* alginate-based gel is a promising candidate for bioprinting, offering a balanced combination of viscosity and elasticity.

In particular, regarding the rheological tests, all the formulated hydrogels show non-Newtonian behavior; in fact, their apparent viscosity (η) is dependent on shear rate ($\dot{\gamma }$). By increasing the shear rates, a clear shear-thinning behavior can be detected, with the viscosity value decreasing at increasing shear rates (Figure 1d). The shear-thinning ability is an important requirement for bioinks to be printable [14] as the reduction in η values improves the bioink process. The hydrogels can be easily extruded as soon as the shear stress exceeds the yield stress, σ_y_ (Table 2). The yield stress parameter reveals the resistance of the fluid to flow during the extrusion process and the capability to support subsequent 3D-printed layers without squeezing [15]. The use of CaCl_2_ improves alginate-based hydrogel mechanical properties, due to a crosslinking reaction between the carboxyl groups of sodium alginate and Ca^2+^ ions. Alginate-10% exhibits solid-like behavior for a wider range of angular frequencies than Alginate-8% ((10 < ω < 100) rad/s and (13 < ω < 100) rad/s, respectively) as can be seen in Figure 2b,c. This evidence suggests selecting Alginate-10% as a more promising candidate bioink to realize scaffolds through the 3D bioprinting process.

Regarding the morphological investigations of the scaffolds, phase-contrast inverted microscopy was employed to monitor and quantify spheroid growth on the 10% alginate scaffolds. On the other hand, observation of the Cellink alginate scaffold was not possible due to its dense structure, which interfered with both visual inspection and image acquisition. The panels shown in Figure 6a illustrate spheroid growth from the day following printing up to day 6, with spheroid areas quantified for analysis. As shown in Figure 6b, statistical analysis revealed a significant increase in spheroid area on days 5 and 6 compared to day 1 for the 10% *w*/*v* alginate. This indicates a positive cellular response and supports the hydrogel’s biocompatibility.

Following DAPI staining, the cells’ nuclei were clearly highlighted due to their fluorescence, confirming the cellular viability of the 3D aggregates. H&E staining revealed that each spheroid was composed of multiple cells in both matrix types (Figure 7c,d), with the nuclei clearly highlighted in purple in the top left corner of the images.

With reference to the FE-SEM observations, the abundant mitochondria observed are consistent with the known ultrastructural features of leiomyosarcoma spheroids, which are typically rich in mitochondria. Leiomyosarcoma, a highly malignant tumor with metastatic potential, is characterized by its smooth muscle tissue origin. The literature reports that smooth muscle tumors, including leiomyosarcoma, frequently show a high density of mitochondria, a feature that has been linked to their energetic demands and aggressive nature. This mitochondrial enrichment is sometimes utilized as a histopathological marker for identifying smooth muscle differentiation in tumor cells [16]. Moreover, the observed disruption of the spheroid surface likely reflects the high metabolic activity and potentially altered cell morphology associated with the malignant nature of the tumor, providing further insight into the tissue architecture at the ultrastructural level. The FE-SEM images of the Cellink bioink show filamentous structures that may resemble collagen fibers. As is well-known, collagen constitutes the extracellular matrix and plays a crucial role in tumor formation and progression [5].

As for the FE-SEM images of the 10% *w*/*v* alginate scaffold, they reveal the presence of subcellular structures that may correspond, on the basis of their small size, to mitochondria, as identified in the commercial matrix. Additionally, the surface of the spheroid shows fibril-like structures, potentially collagen fibers. The collagen fibers are especially noticeable in the regions surrounding the spheroid, suggesting a possible interaction between the cells and the extracellular matrix.

Furthermore, the comparison between the two scaffolds highlights significant differences in their mesh structures. The 10% *w*/*v* alginate scaffold presents a more tightly organized mesh, while the commercial matrix displays a relatively looser, more interconnected network. These differences may influence the mechanical properties and the behavior of the encapsulated cells, potentially affecting their proliferation and interaction with the scaffold.

In comparison to previous alginate-based bioprinting studies, especially those by Tabriz et al. [2] and Siviello et al. [8], the present work introduces several distinctive aspects. While Tabriz et al. focused on fabricating complex 3D alginate structures using a multi-step crosslinking strategy optimized for tissue engineering and vascular constructs, our study emphasizes the development of a simplified, lab-formulated alginate bioink tailored for efficient tumor spheroid formation in vitro. Furthermore, Siviello et al. investigated the viscoelastic aging behavior of alginate gels, whereas our work integrates rheological optimization with practical bioprinting performance and biological validation in the context of 3D cancer cell culture. Primarily, our approach leverages a cost-effective and customizable formulation, avoiding the constraints of commercial bioinks and enabling on-demand preparation for specific research needs. In addition, by using a single post-printing crosslinking step and a commercially available pneumatic extrusion bioprinter, we propose a reproducible and accessible workflow suitable for standard laboratory settings. Finally, we demonstrate that the proposed bioink effectively supports sustained spheroid growth and biological activity, which is essential for modeling tumor cell behavior and was not a primary focus of previous studies.

## 5. Conclusions

### 5.1. Cost-Effectiveness and Ethical Benefit

The use of low-cost, in-house-formulated bioinks presents substantial advantages for modeling tumor cell growth in vitro. These bioinks facilitate the creation of models that more accurately replicate the cellular microenvironment, eliminating the need for expensive materials typically used in tissue engineering applications. This approach is particularly advantageous for tumor cell studies, where the focus lies on understanding fundamental cellular behaviors rather than on regenerating tissues. By enabling the production of relevant experimental models at a reduced cost, this strategy enhances research efficiency without compromising scientific rigor.

In addition, using bioinks free from animal-derived components offers important ethical benefits. By circumventing the need for animal tissues, this approach aligns with the growing commitment to reduce animal testing in research, providing a more ethical and sustainable alternative for in vitro studies.

### 5.2. Biological Validation of the Lab-Prepared Bioink

In this study, our lab-prepared alginate hydrogel demonstrated favorable rheological and morphological properties and effectively supported cell viability and proliferation over time, as confirmed by phase-contrast microscopy and DAPI staining. After 72 h, microscopy revealed consistent and increasing cell presence, validating the bioink’s capacity to sustain viable and metabolically active cells within the 3D matrix. These observations confirm the biological compatibility of the formulation and its potential to support the development of functional tissue models.

### 5.3. Flexibility and Reproducibility

Economically, the lab-prepared bioink offers substantial advantages over commercial alternatives. Producing the bioink from accessible raw materials provides greater control over formulation, significantly reducing costs while enabling on-demand customization. Additionally, this flexibility allows researchers to adapt the bioink composition to specific experimental needs. However, it should be acknowledged that achieving batch-to-batch reproducibility may require further optimization and standardization.

In terms of economic comparison, the raw material cost for preparing the lab-formulated alginate bioink is approximately €0.05–0.10 per milliliter, including sodium alginate and crosslinker. The commercial Cellink Bioink is approximately 400 to 800 times more expensive per milliliter than the lab-prepared sodium alginate-based bioink, further underscoring the significant economic advantage of the proposed lab-formulated bioink for research applications. This represents a cost reduction of over 99%, highlighting the substantial economic advantage of the in-house formulation. Such a difference is particularly relevant for research contexts that require large volumes of bioink or iterative experimental work, where material costs can become a limiting factor.

### 5.4. Comparison with Commercial Bioinks

It is also important to note that the commercial bioink used for comparison is proprietary, and its exact composition is not disclosed. Unknown additives, viscosity modifiers, or extracellular matrix components in the commercial product may significantly influence its mechanical behavior and cellular response. This lack of transparency limits the possibility of making a fully direct and quantitative comparison between the two materials. In contrast, our fully characterized, lab-prepared alginate hydrogel offers a customizable, low-cost, and reproducible platform that can be tailored to specific research needs and biomedical applications.

### 5.5. Study Limitations and Future Directions

Some limitations of this study must be acknowledged. The biological validation was performed using a single cancer cell line (SK-LMS-1), which restricts the generalizability of the findings. Future studies should aim to test the bioink with additional cell types, including non-cancerous and primary cells, to further demonstrate its versatility and relevance across different biological contexts.

### 5.6. Conclusion

In summary, although further validation is required, the developed bioink demonstrates strong potential as a cost-effective, ethically conscious, and customizable material for 3D bioprinting applications in cancer research. It represents a viable and sustainable alternative to commercial bioinks, particularly in research settings focused on studying cellular behavior rather than tissue engineering or regeneration.

## Figures and Tables

**Table 1:** Parameters selected for the bioprinting process.

Type of Bioink	Sodium Alginate
Nozzle diameter (µm)	410
Nozzle height (mm)	9.7
Print speed (mm/s)	2
Air pressure (kPa)	49
Printhead T (°C)	24

**Table 2:** Rheological characteristics of bioinks at 25 °C.

Formulations	G′ (Pa) @ 1 rad/s	G″ (Pa) @ 1 rad/s	G′ (Pa) @ 100 rad/s	G″ (Pa) @ 100 rad/s	σ_y_ (Pa)	η_y_ (Pa∙s)
Reference Bioink	6584 ± 20	1410 ± 30	10,826 ± 36	2358 ± 26	71 ± 2	688 ± 25
Alginate 8	157 ± 9	297 ± 6	3057 ± 41	1739 ± 20	84 ± 15	863 ± 156
Alginate 10	251 ± 16	432 ± 26	4002 ± 120	2071 ± 82	147 ± 15	1500 ± 171

## Data Availability

The original contributions presented in this study are included in the article. Further inquiries can be directed to the corresponding authors.
